# Structural Connectivity in Down Syndrome and Alzheimer’s Disease

**DOI:** 10.3389/fnins.2022.908413

**Published:** 2022-07-22

**Authors:** Fedal Saini, Flavio Dell’Acqua, Andre Strydom

**Affiliations:** ^1^Department of Forensic and Neurodevelopmental Sciences, Institute of Psychiatry, Psychology and Neuroscience, King’s College London, London, United Kingdom; ^2^Department of Neuroimaging, Institute of Psychiatry, Psychology and Neuroscience, King’s College London, London, United Kingdom; ^3^Department of Forensic and Neurodevelopmental Sciences, Sackler Institute for Translational Neurodevelopment, Institute of Psychiatry, Psychology and Neuroscience, King’s College London, London, United Kingdom

**Keywords:** down syndrome, trisomy 21, Alzheimer’s disease, white matter, structural connectivity, diffusion tensor imaging (DTI), tract based special statistic (TBSS), functional connectivity

## Abstract

Down syndrome (DS) arises from the triplication of chromosome 21, which leads to an atypical neurodevelopment and the overproduction of the amyloid precursor protein, predisposing to early Alzheimer’s disease (AD). Not surprisingly, trisomy 21 is widely considered a model to study predementia stages of AD. After decades, in which neural loss was the main focus, research in AD is now moving toward understanding the neurodegenerative aspects affecting white matter. Motivated by the development of magnetic resonance imaging (MRI)-based diffusion techniques, this shift in focus has led to several exploratory studies on both young and older individuals with DS. In this review, we synthesise the initial efforts made by researchers in characterising *in-vivo* structural connectivity in DS, together with the AD footprint on top of such pre-existing connectivity related to atypical brain development. The white matter structures found to be affected in DS are the corpus callosum and all the main long-association fibres, namely the inferior fronto-occipital fasciculus, the inferior and superior longitudinal fasciculus, the uncinate fasciculus and the cingulum bundle. Furthermore, the cingulum bundle and the corpus callosum appear to be particularly sensitive to early AD changes in this population. Findings are discussed in terms of their functional significance, alongside methodological considerations and implications for future research.

## Introduction

Down syndrome (DS), also referred to as trisomy 21, is the most common genetic cause of intellectual disability, occurring in about 1 out of 700 live births worldwide (Parker et al., 2010). The aetiology of DS is well known and consists of a third copy of chromosome 21, with exceptions being mosaicism and translocation, which account for about 5% and 2% of the total cases, respectively (Antonarakis et al., 2020). Down syndrome is characterised by atypical development of motor (i.e., low muscle tone and poor postural control) and cognitive functions (mainly hippocampal-, fronto- and cerebellar-dependent functions).

The impact of trisomy 21 on the central nervous system unfolds throughout life, leading to mild-to-moderate intellectual disability in childhood and adolescence. Despite inter-individual variability, the late adulthood of most individuals with DS is characterised by a progressive loss of cognitive abilities associated with the development of Alzheimer’s disease (AD). Indeed, due to the Aβ-amyloid precursor protein gene (APP; the same gene implicated in familial AD) triplication in chromosome 21, amyloid deposition can be found in virtually all individuals with DS by their third decade (Davidson et al., 2018), several years earlier than the pathology development in the general population (sporadic Alzheimer disease, sAD). Nevertheless, the AD clinical manifestation appears later in life, affecting about 88% of the population with DS by the age of 65 (McCarron et al., 2017).

Despite brain investigation on DS being a complicated task, several studies have explored the neural configuration in people with DS throughout development, giving rise to increasing, although far from conclusive, accumulation of knowledge. Whole-brain voxel-based morphometry analyses of magnetic resonance imaging (MRI) revealed a consistent pattern of grey matter volume loss, involving mainly the fronto-temporal areas, structures of medial temporal lobes (e.g., hippocampus) and the cerebellar regions, in both children (Menghini et al., 2011; Carducci et al., 2013; Lee et al., 2015; Zhang et al., 2019) and adults with DS as compared to the age-matched healthy controls (White et al., 2003; Teipel et al., 2004; Annus et al., 2017; Bletsch et al., 2018; Pujol et al., 2018).

The development of MRI-based diffusion techniques over the past few decades has allowed researchers to consider the importance of white matter microstructural integrity in the characterisation of AD progression. This shift in focus is gradually being employed also in the DS research field, with several recently published studies. Therefore, the current work aims to provide a review of the structural white matter neuroimaging literature in DS. The aim is to synthesise the initial findings in characterising *in-vivo* structural connectivity in DS, together with the AD footprint on pre-existing atypical development of connectivity structures. The findings from other neuroimaging techniques, such as resting-state fMRI (rs-fMRI), will also be integrated to support hypotheses regarding functional mechanisms. Future directions for research will be discussed.

## Volumetric Magnetic Resonance Imaging and Diffusion Magnetic Resonance Imaging

Volumetric MRI techniques can provide the total brain’s white matter volume which, in the population with DS, appears to be generally reduced (Pinter et al., 2001). Such white matter volume reduction is characterised by a frontal predominance, extending further to the temporal and parietal areas, as well as the cerebellum and cingulate cortex (Pinter et al., 2001; Menghini et al., 2011; Carducci et al., 2013; Bletsch et al., 2018). However, volumetric MRI techniques can provide only a measure of the gross degree of white matter degeneration, a phenomenon that is likely chronologically subsequent to more subtle aberrations in microstructural integrity.

To obtain a more detailed measure of white matter microstructural properties, diffusion imaging techniques can be employed. Diffusion imaging is an MRI-based technique that is used to study the brain’s white matter microstructure integrity *in-vivo*. From the exploitation of water diffusion quantification in biological tissues, diffusion tensor imaging (DTI) provides measures of magnitude, degree and orientation of diffusion anisotropy as output metrics (Basser et al., 1994). Diffusion parameters commonly used are fractional anisotropy (FA), mean diffusivity (MD), as well as axial and radial diffusivity (see Table 1). FA informs about the degree of diffusion directionality (i.e., diffusion being not uniform along every direction), while MD is a direction-independent measure of the magnitude of diffusion itself (i.e., the average degree of hindrance and restriction experienced by water molecules within the biological tissue). Finally, axial and radial diffusivity measure, respectively, the diffusion of water along and perpendicular to the main axis of the diffusion tensor, which approximates the main orientation of the underlying white matter (Pierpaoli and Basser, 1996; Solowij et al., 2017). Together these metrics can effectively probe the microstructural characteristics of white matter and extract information about axonal density, fibre coherency and myelinisation level (for a discussion about the diffusion metrics, see Jones et al., 2013). Therefore, in the case of the pathological processes associated with AD, where volumetric MRI measures the gross neural features that manifest late in the neurodegenerative process, diffusion imaging can potentially inform about the white matter microstructural integrity at earlier stages of AD.

**TABLE 1:** Main characteristics and interpretations of diffusion magnetic resonance imaging (MRI) metrics.

Name	Measure	Interpretations
Fractional anisotropy	Rotationally invariant index of the degree of microstructural organisation and diffusion directionality	Reduced fractional anisotropy might reflect ● damage or degeneration to axons or myelin ● reduced axonal packing density ● reduced axonal directional coherence
Mean diffusivity	Rotationally invariant index of the average diffusivity or water mobility within each image voxel	Increased mean diffusivity might reflect ● reduced white matter density due axonal degeneration ● increased extracellular space due to myelin degeneration ● decreased axonal packing
Axial diffusivity	Rotationally invariant index of diffusivity computed along the direction of maximum diffusivity of the diffusion tensor	Reduced axial diffusivity might reflect ● axonal injury/degeneration ● increased tortuosity ● less coherent axons orientation
Radial diffusivity	Rotationally invariant index of diffusivity computed perpendicularly to the direction of maximum diffusivity of the diffusion tensor	Increased radial diffusivity might reflect ● myelin loss ● axons loss or decrease axonal density ● reduced axonal packing ● increased extra-axonal space

To date, only a few studies have investigated white matter integrity in DS using diffusions techniques, producing nine cross-sectional investigations (see Table 2); three studies have compared DS children, toddlers and young adults with age-matched healthy controls (Gunbey et al., 2017; Romano et al., 2018; Lee et al., 2020), while four studies concentrated on the same comparison in adults with DS (Fenoll et al., 2017; Koenig et al., 2019; Patrick et al., 2019; Bazydlo et al., 2021). Finally, three studies (Powell et al., 2014; Rosas et al., 2020; Bazydlo et al., 2021) have compared both DS adults without and with AD (DsAD) with healthy controls. These studies have been identified through a literature search using the following databases up to December 2021: MEDLINE, Embase and psycINFO. The search terms used were “Down syndrome,” “trisomy 21,” “white matter,” “structural connectivity,” “diffusion MRI,” “DTI,” “TBSS” and “fractional anisotropy.” Only studies that included individuals with DS and that employed diffusion-based MRI techniques were included. Finally, the bibliographies of included studies were searched for additional references.

**TABLE 2:** Principal characteristic and main results of diffusion imaging studies in Down syndrome.

Paper	Groups	Sample *n*	Sample age	Main results
			(Mean; SD)	
Gunbey et al. 2017	DS	10	2,6; ± 0,69	*Voxel-wise TBSS analysis (DS > HC)*
				● FA decrease of IFOF, ILF, UF, right cerebral peduncle, corpus callosum body, right anterior limb of internal capsule.
				● MD increase of right IFOF, right ILF, UF, ATR, right cerebral peduncle, left external capsule, left anterior thalamic radiation, anterior corona radiata.
				*ROI analyses (DS > HC)*
				● Lower FA in right cerebellar peduncle, right ILF, UF.
	HC	10	2,5; ± 0,7	● Volumetric reduction of corpus callosum and right cerebellar white matter in DS as compared to HC.
Lee et al. 2020	DS	15	17,0; ± 5.5	*Voxel-wise TBSS analysis (DS > HC)*
				● FA decrease in only 1% of total voxels: cerebellar (inferior and middle) and cerebral peduncles, external and internal capsule, anterior corona radiata, fornix, and medial lemniscus. No difference in MD.
				*TBM analyses (DS > HC)*
				● Hypoplasia of several areas (cerebellar area, pons, and fornix).
				*ROI analysis (DS > HC)*
				● Hypoplasia transverse pontine fibres, cerebellar and cerebral peduncles, fornix, frontal white matter, anterior limb of internal capsule, and cingulum.
				*Tractography analysis (passing though most affected ROIs) with continuous negative Ln-J voxel (DS)*
	HC	15	17,8; ± 6,1	● Hypoplasia of the fronto-pontine-cerebellar and temporo-occipito-parietal-pontine-cerebellar pathways, as well as of the fibres connecting the olives to the contralateral cerebellar cortex (thought he inferior cerebellar peduncles).
Romano et al., 2018	DS	17	23,7; (range 15–27)	*Voxel-wise TBSS analysis (DS > HC)*
				● FA decrease of IFOF, ILF, ATR, CST.
	HC	17	21,6; (range 14–27)	● MD, Axial and Radial diffusivity increase of IFOF, ILF, UF, Cingulum, ATR, CST, Splenium of corpus callosum, as well as MD and Axial diffusivity increase of SLF.
Koenig et al., 2019	DS	10	29,4; (range 26–32)	*Voxel-wise volumetric MRI analyses (DS > HC)*
				● Reduced WM volume in bilateral ACC and PCC.
				*Diffusion ROI analysis (DS > HC)*
				● Radial diffusivity in right IPL and right precuneus was related with attention scores in DS.
	HC	10	28,7; (range 24–33)	● Radial diffusivity in right rostral MFG, right ACC and left PCC was related with plasma inflammatory markers.
Fenoll et al., 2017	DS	45	35,3; (range 18–52)	*Voxel-wise TBSS analysis (DS > HC)*
				● FA reduction in frontal lobes, semiovale centrum, corpus callosum, external capsule, internal capsule, putamen, thalamus, pyramidal tracts, brainstem. Alterations were more severe in the frontal-subcortical circuits.
	HC	45	34,6; (range 19–51)	● Positive correlation between FA and semantic fluency in several regions (frontal lobes, corpus callosum, semioval centres, arcuate fasciculus, caudate nucleus, external capsule, thalamus, and hippocampus).
Patrick et al. 2019	DS	25	–	*Voxel-wise TBSS analysis (DsAD > DS)*
	DsAD	8	–	● MD increase of ILF, SLF, corona radiata, and fronto-occipital fasciculus.
Bazydlo et al. 2021	DS	46	39,1; ± 7,7	*Voxel-wise TBSS analysis and Pearson’s partial correlation analyses (across all groups)*
				● FA in SLF and ILF positively correlates with measures of episodic memory.
				● MD in SLF and ILF negatively correlates with measures of episodic memory.
				● The results didn’t change after removing the participants with preclinical/prodromal AD and AD from the analyses.
	DS preclinical/prodromal AD	3	45,5; ± 3,5	
	DsAD	3	50,5; ± 5,2	
Powell et al. 2014	DS	10	50,6; ± 5,5	*Voxel-wise TBSS analysis (DS > HC)*
				● FA reduction of IFOF, ILF, SLF, UF, Cingulum, Splenium of corpus callosum, Thalamic Radiation. The largest number of significantly lower FA voxels were found in the frontal lobes. Positive correlation between FA and frontal executive function in several areas.
	DS preclinical/prodromal AD	10	52,1; ± 7,5	*Voxel-wise TBSS analysis (DsAD > DS)*
	HC	10	51,7; ± 2.1	● No statistically significant results after multiple comparison correction.
Rosas et al. 2020	DS	11	48.5; ± 6.1	*Voxel-wise TBSS analysis (DsAD > Ds with preclinical/prodromal AD > DS)*
				● Average FA values tend to decrease (as a trend) in the progression from DS to DS with preclinical/prodromal AD to DsAD in Genu, Splenium, Cingulum bundle, UN, and ILF. Diffusion metrics of Genu, Splenium and Cingulum bundle correlates with measure of general cognitive functioning and memory across groups.
				*Voxel-wise TBSS analysis (Ds* with *preclinical/prodromal AD > DS)*
				● FA decrease of Genu, Splenium, Cingulum bundle, ILF in DS with preclinical/prodroma AD as compared to DS.
				*Voxel-wise TBSS analysis (DsAD > DS)*
				● FA decrease and MD, Axial and Radial diffusivity increase Genu, Splenium, Cingulum bundle, ILF, UN, SLF, Cingulum angular bundle in DsAD as compared to DS.
	DS preclinical/prodromal AD	12	51.5; ± 5.2	*Partial Spearman correlation analysis between ROI’s FA and amyloid SURV scaling (across all groups*)
	DsAD	8	54.3; ± 7.7	● Low Genu FA correlate with amyloid burden in frontal region and low Splenium FA correlate with amyloid burden in precuneus and parietal regions. Low Cingulum bundle FA correlate with amyloid burden in entorhinal cortex and parietal cortical region.

*DS, Down syndrome; DsAD, Down syndrome with Alzheimer disease; HC, healthy control; SD, standard deviation; TBSS, tract-base spatial statistic; ROI, region of interest; FA, fractional anisotropy; MD, mean diffusivity; SURV, standardised uptake value ratio; Ln-J, Logarithm of the determinant of the Jacobian; IFOF, inferior fronto-occipital fasciculus, ILF, inferior longitudinal fasciculus; SLF, superior longitudinal fasciculus; UF, uncinate fasciculus; ATR, anterior thalamic radiation; CST, corticospinal tract.*

Findings from these studies will be presented in the following sections and will be grouped based on the main white matter categorisation, which broadly divides the brain’s tracts into three main groups based on the directionality of their connectivity: association, commissural and projection fibres. While association fibres connect cortical areas within the same hemisphere, commissures provide a structural connection between brain hemispheres. Finally, projection fibres connect cortical areas with the rest of the neuroaxis (Catani and De Schotten, 2008). The cingulum bundle (which belongs to the association fibres) will be discussed separately to highlight its relevance in DS.

## The Long-Association Fibres

**FIGURE 1:**
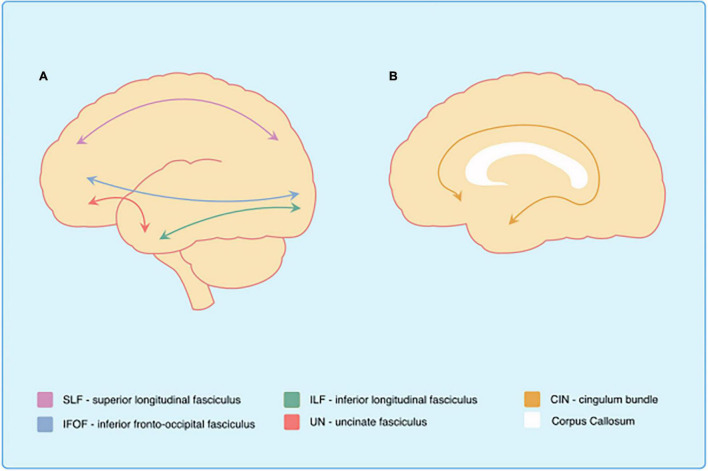
White matter tracts affected in Down syndrome. Schematic representation of the main white matter structures affected in Down syndrome. Al Sagittal view of the brain. Bl Midsagittal view of the brain.

**FIGURE 2:**
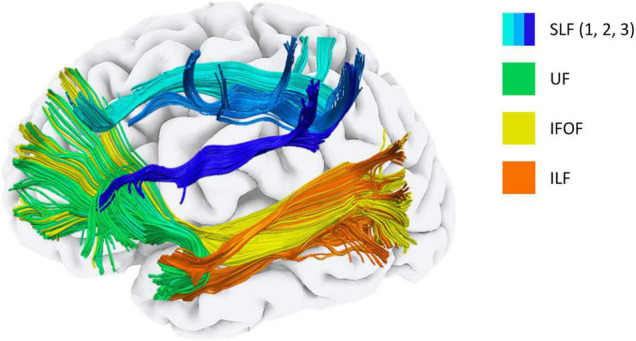
Main long association fibres affected in Down syndrome. Tractography representation of the main long association fibres affected in Down syndrome. SLF, Superior longitudinal fasciculus; IFOF, Inferior fronto-occipital fasciculus; UF, Uncinate fasciculus; ILF, inferior longitudinal fasciculus.

**FIGURE 3:**
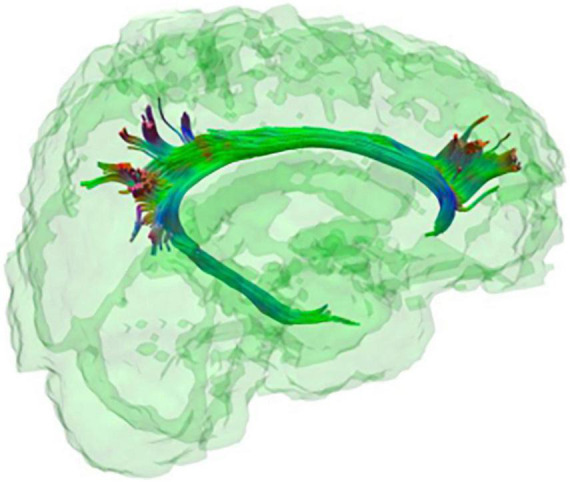
The cingulum bundle. Tractography representation of the cingulum bundle.

**FIGURE 4:**
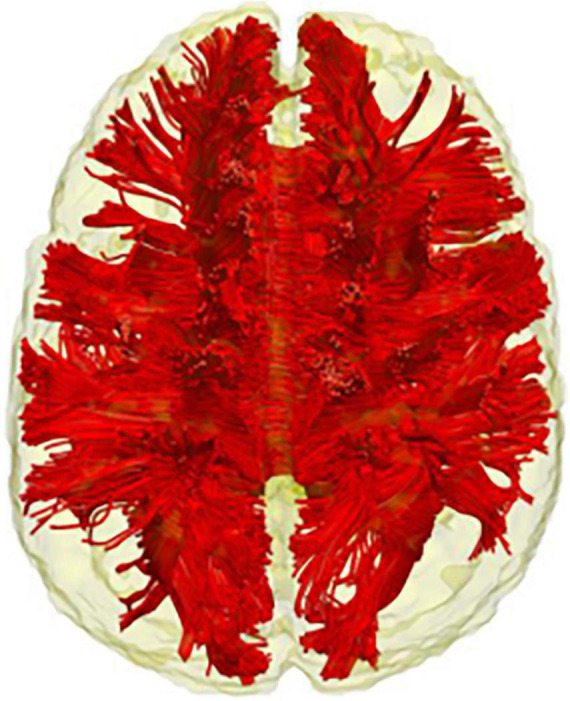
The corpus callosum. Tractography representation of the corpus callosum.

Association fibres are white matter formations interconnecting brain regions that lie within the same hemisphere. Anatomically speaking, association fibres are divided into two typologies: short-association fibres connecting adjacent cerebral gyri and long-association fibres connecting relatively distant brain areas and lobes (Catani and De Schotten, 2008). Reduced FA together with increased MD, axial and radial diffusivity of all the main long-association fibres have been reported in most diffusion imaging investigations in individuals with DS. Specifically, the structural loss affects the superior longitudinal fasciculus (SLF), inferior longitudinal fasciculus (ILF), inferior fronto-occipital fasciculus (IFOF), uncinate fasciculus (UF) and the cingulum bundle (see Figures 1–4 and Table 2). These findings have been reported in almost all the possible comparisons; children (Gunbey et al., 2017) and adults with DS (Powell et al., 2014; Romano et al., 2018; Bazydlo et al., 2021) as compared to the age-matched controls from the general population, as well as additional reductions in DsAD as compared to healthy DS individuals (Patrick et al., 2019; Rosas et al., 2020). In terms of clinical significance, FA reductions in all these tracts were associated with poorer performance in verbal comprehension and motor coordination tasks (Powell et al., 2014), while reduced FA and increased MD in the ILF and SLF were associated with decreased episodic memory ability (Bazydlo et al., 2021).

Understanding the functional significance of such structural atypicality is a complicated task, as these bundles represent the entire inter-hemispheric wiring of the brain. The parieto-occipital regions are connected to the frontal lobes by means of the IFOF (Wu et al., 2016) and to the temporal poles *via* the ILF (Herbet et al., 2018). The IFOF has been associated with language processing and transmission (Duffau, 2008; Almairac et al., 2015), while the ILF plays a role in lexical and semantic processing (Herbet et al., 2018). The UF is a white matter bundle that connects the temporal poles with the orbitofrontal cortex (Catani et al., 2002) and is involved in episodic memory formation and retrieval (Squire and Zola-Morgan, 1991; Nestor et al., 2004). Finally, the SLF connects the inferior parietal lobule with the frontal cortex and is considered a key structure in the visuospatial integration and motor planning functions (Rushworth et al., 2006; De Schotten et al., 2011).

The IFOF, ILF and UF (together with the middle longitudinal fasciculus) form the connective structure of the anterior temporal network, also referred to as the auditory ventral stream, one of the key networks involved in lexical and semantic processing (Duffau et al., 2013; Catani and Dawson, 2017). Therefore, the microstructural loss affecting these white matter bundles could be related to the language and verbal memory impairments commonly observed in DS (Grieco et al., 2015). Such a conclusion would also be supported by the association of FA reduction in these areas with measures of verbal comprehension (Powell et al., 2014). However, at present, this hypothesis has not been directly tested, therefore lacking empirical support.

The structural integrity loss of all the main long-association fibres potentially implies that the communication between distal brain regions (within the same hemisphere) would be impaired in this population. Consequently, the capacity to transmit information through the whole brain would be altered. Support for this idea comes from an rs-fMRI study, in which, decreased “global” functional connectivity (i.e., the functional connectivity between relatively distant brain regions) was observed in adults with DS as compared to healthy controls (Anderson et al., 2013). Direct associations between long-association fibres integrity and global functional connectivity have never been directly investigated; nevertheless, speculations can be made. When considered together, this evidence may suggest that long-association fibres’ integrity loss might contribute to the reduction in functional connectivity between the brain’s distal areas observed in DS. On the other hand, a heightened “local” functional connectivity (i.e., the functional connectivity between short-distance brain regions) was observed in the very same population (Anderson et al., 2013). This latter finding aligns with the observation that short-distance association fibres are spared in DS. This increase in local functional connectivity could be considered a compensatory mechanism, counterbalancing the decreased global connectivity. Finally, integrity loss of the long-association fibres could also explain the fragmented and overtly simplified brain network parcellation reported in DS (Anderson et al., 2013; Vega et al., 2015; Figueroa-Jimenez et al., 2021).

Despite the different functional interpretations that might be drawn, it is noteworthy that all the findings regarding long-association fibres found to be involved in DS also appear to be affected in sAD (Stricker et al., 2009; Taoka et al., 2009; Bosch et al., 2012; Huang et al., 2012). The majority of diffusion imaging studies described so far employed DS individuals without AD, and yet their white matter structural appearance closely resembles that of sAD—the literature on diffusion changes in sAD support a common and widespread range of white matter structural abnormalities, which are generally reported as integrity loss in all the long association fibres, amongst the others (Stricker et al., 2009; Agosta et al., 2011; Alves et al., 2012; Bosch et al., 2012; Huang et al., 2012). One interpretation of the similarities between DS and sAD is that the white matter degeneration and demyelination processes may already begin in early adulthood and unfold gradually throughout adult life in the population with DS, meaning that MRI features associated with sAD may be noted in younger “healthy” individuals with DS. Diffusion imaging studies that compared DsAD with healthy DS reported FA reductions and MD increases in the IFOF, ILF, UN, SLF and cingulum in DsAD participants (Powell et al., 2014; Patrick et al., 2019; Rosas et al., 2020). These data suggest that long-association fibres might be affected in both pre- and post-AD life stages in this population, with further changes during the development of AD pathology.

### The Cingulum Bundle

A cingulum bundle is a heterogeneous group of white matter fibres that forms a near-complete ring from the orbito-frontal cortex to the temporal lobe (see Figures 1, 3), encompassing the dorsal corpus callosum (Schmahmann et al., 2007). The cingulum bundle interconnects medial temporal, parietal and frontal cortices, while also connecting the cingulate gyrus with subcortical nuclei (Bubb et al., 2018). The reduced structural integrity of the cingulum bundle can be observed in toddlers and adults with DS as compared to healthy controls (Powell et al., 2014; Romano et al., 2018; Lee et al., 2020; Rosas et al., 2020) and in DsAD as compared to healthy DS (Rosas et al., 2020; see Table 2). This suggests the presence of an incremental microstructural alteration that affects the cingulum bundle, with its origins in the developmental stages of DS, which is further impacted by AD. Moreover, the cingulum bundle is one of the few white matter structures in which FA values appear to be sensitive during early neurodegenerative changes in DS, which is able to discriminate between DS and DS with preclinical/prodromal AD (referred to as DS with mild cognitive impairment by Rosas et al., 2020). Finally, FA reduction in the cingulum bundle has been associated with measures of memory and verbal learning, as well as with high-amyloid burden in parietal cortical regions and in the entorhinal cortex in DS (Rosas et al., 2020), one of the most sensitive regions in sAD (for a review see Pini et al., 2016).

By integrating the evidences from the rs-fMRI and volumetric MRI fields, the cingulum bundle provides the physical connection of the posterior node of the Default mode network (DMN) with its frontal and temporal counterparts (Greicius et al., 2003; Schmahmann et al., 2007). The DMN is one of the most widely studied intrinsic brain networks, which displays high levels of metabolic activity during resting-state conditions in healthy individuals (Gusnard and Raichle, 2001; Fox et al., 2005; Buckner et al., 2009). Reduction of the DMN posterior node functional connectivity is a common feature of sAD (Jacobs et al., 2013; Badhwar et al., 2017). Interestingly, the same result has been recently demonstrated in DsAD as well (Wilson et al., 2019). Therefore, it would be reasonable to assume that the integrity loss of the cingulum bundle, so frequently reported by diffusion studies in DS, could play an important role in the DMN connectivity alterations. However, more studies targeting this specific hypothesis are needed.

The importance of the cingulum bundle is further evincible when the rs-fMRI studies are interpreted in light of volumetric MRI literature results; the posterior cingulate/precuneus complex represents the neural substrate of the DMN posterior node (Fransson and Marrelec, 2008; Andrews-Hanna et al., 2014). The posterior cingulate/precuneus complex has been disproportionally represented as a site of sAD pathology in neuroimaging studies (Takahashi et al., 2002; Zhang et al., 2007; Alves et al., 2012; Bosch et al., 2012; Hillary and Grafman, 2017), and might constitute a network-based biomarker in sAD (Jacobs et al., 2013). It is noticeable that when individuals with DS develop AD, the posterior cingulate/precuneus complex is one of the most affected brain areas in terms of volume loss (Beacher et al., 2009; Sabbagh et al., 2015; Annus et al., 2017; Lao et al., 2017). Furthermore, a longitudinal investigation of DS found the precuneus to be the only cortical volume negatively correlated with global cortical amyloid deposition (Lao et al., 2017). It is therefore interesting to note how three different types of data (volumetric MRI, diffusion MRI and rs-fMRI) converge in suggesting a complex interaction of volumetric, functional and structural connectivity loss involving the DMN posterior node or posterior cingulate/precuneus and the cingulum bundle. In conclusion, these cross-methodological comparisons suggest that the cingulum bundle is a structure of primary importance when attempting to understand the link between sAD and DsAD and the development of AD in the context of the DS neurodevelopment.

## Commissural Fibres

The white matter structures mentioned so far are all long-association fibres supporting the brain’s intra-hemispheric communication. However, information transmission in the brain is also possible between hemispheres and is supported through the commissural system. The corpus callosum is the largest brain commissure, interconnecting several brain areas (Raybaud, 2010; see Figure 1), and although no clear boundaries are observable, it is commonly subdivided into genu, body and splenium (Witelson, 1989). Reduced FA has been observed in the corpus callosum of children and adults with DS as compared to the age-matched healthy controls (Powell et al., 2014; Fenoll et al., 2017; Gunbey et al., 2017; see Table 2). Teipel and Hampel (2006) hypothesised that the corpus callosum structural loss should be treated as a cognitive biomarker in DS. Indeed, they found an association of corpus callosum atrophy with general cognitive, as well as language, orientation, visuospatial and memory performance in adult participants with DS (Teipel S. J. et al., 2003). They also showed that the extent of corpus callosum atrophy in elderly individuals with DS (affecting mainly the splenium) is comparable to that of the hippocampus. Further support for this idea comes from a recent study by Rosas et al. (2020), in which a group of adults with DS was compared with both DsAD and DS with preclinical/prodromal AD. Their results showed a gradual progression of the diffusion metrices from healthy DS to DS with preclinical/prodromal AD and from DS preclinical/prodromal AD to DsAD in the genu and splenium of corpus callosum (together with the cingulum bundle, ILF and UN). Interestingly, not only the splenium was associated with memory performance, but it was the white matter tracts that differed significantly between DS with preclinical/prodromal AD and healthy DS, and therefore considered sensitive to AD-like early changes in this population. Given that the corpus callosum represents one of the biggest connective structures of the brain, it is difficult to precisely outline what could be the functional counterpart of the structural damages observed in all these studies. Once again, drawing from different research fields might prove helpful in disentangling the role of the corpus callosum in DS cognition and the advent of AD.

Resting-state functional connectivity studies have reported a reduction (or near-total absence) of anti-correlations in both young DS and DsAD as compared to the age-matched healthy controls (Anderson et al., 2013; Wilson et al., 2019). The same phenomena can also be observed in the population with sAD (Wang et al., 2007; Weiler et al., 2017). While positive correlation indicates that two regions are comodulated (either during a task or at rest), a negative or *anti*-correlation between networks (or regions) means that the networks are temporally modulated in opposite directions (Fox et al., 2006). Anti-correlation in the brain could represent a “division of labour” between networks with seemingly opposite functions (Fransson, 2006), with failures in such processes being associated with negative cognitive and behavioural outcomes (Weissman et al., 2006; Kelly et al., 2008). In this context, the integrity loss of corpus callosum, commonly observed in DS and DsAD, might potentially be implicated. Although the precise processes involved are still a matter of debate (for a review, see van der Knaap and van der Ham, 2011), the corpus callosum is a fundamental structure for the brain’s functional specialisation (or lateralisation). It is therefore not surprising that individuals with DS exhibit atypical linguistic lateralisation, which might seemingly account for their idiosyncratic verbal profile (Heath et al., 2005; Shoji et al., 2009). Therefore, it could be hypothesised that the profound functional network organisation disruption in DS, evidenced by the lack of anti-correlations, could depend, at least in part, on callosum-driven mechanisms. However, studies targeting the association between corpus callosum integrity and functional anti-correlation are needed to support these speculations.

## Projection Fibres

The third and last type of white matter tracts is projection fibres, a group of both ascending and descending tracts that connect the cortex with subcortical structures, such as deep grey nuclei, brainstem, cerebellum, and spinal cord. Projection fibres mainly carry sensory and motor information and their anatomical nomenclature changes along their course throughout the nervous system. Within the cerebral hemisphere, the projection fibres assume a fan-like shape called corona radiata, which connects the cortical surface with the thalamus (Catani and De Schotten, 2012). Different studies reported reductions in FA and increases in MD of the anterior thalamic radiation in all age groups of individuals with DS, including DsAD, when compared to age matched controls (Powell et al., 2014; Gunbey et al., 2017; Romano et al., 2018; Lee et al., 2020; see Table 2). The anterior thalamic radiation represents the most anterior part of the corona radiata and connects the frontal and cingulate cortex to the thalamic nuclei (Mamah et al., 2010). These structural connectivity findings in DS have been interpreted as a potential sign of the attentional and general executive function deficits associated with DS (Romano et al., 2018). In addition, it has been hypothesised that alterations in the anterior thalamic radiation might disrupt the monoaminergic communication between frontal areas and memory limbic structures, such as the amygdala, resulting therefore in cognitive, personality and behavioural changes frequently observed in DS adults as early dementia symptoms (Ball et al., 2008; Dekker et al., 2015; Ismail et al., 2016).

Moving downward, the neural axis, the thalamic radiations and the corona radiata as a whole pass through the internal capsule and continue inferiorly in the midbrain forming compact bundles, commonly referred to as the cerebral peduncles. At this level, the cerebellar peduncles, connecting the cerebellum with the rest of the brain, can also be found. Finally, the cerebral peduncles of the brainstem converge inferiorly into the spinal cord (Catani and De Schotten, 2012). Interestingly, the internal capsule (especially the anterior limb) and the cerebral peduncles manifest lower FA values and volume reduction in children and toddlers with DS when compared to healthy controls (Gunbey et al., 2017; Lee et al., 2020). Specifically, remarkable hypoplasia was observed in the medial and lateral aspects of the cerebral peduncles, while the intermediate part did not differ from healthy controls. The intermediate aspect of the cerebral peduncles hosts the corticospinal tract (or pyramidal tract), a group of axons carrying motor information from the cerebral cortex to the spinal cord (Kwon et al., 2011). This finding led Lee et al. (2020) to conclude that the corticospinal tract is relatively spared in DS. They suggested that the motor impairment frequently observed in this population may be explained by the integrity loss observed in the afferent cerebellar peduncles and the cerebellum. This conclusion was further supported by the results of the tractography analyses. The authors employed a novel diffusion imaging technique called tensor-based morphometry, which identifies regional structural (volumetric) differences by measuring the degree of nonlinear deformation required to align each subject’s data to a common anatomical template (Lee et al., 2020). Seeding tractography in the hypoplastic cerebellar peduncles regions, the deterministic tractography was able to reconstruct white matter trajectories associated with volume loss. The results evidenced severe hypoplasia in motor-control relevant tracts, such as the pontine-cerebellar and olivo-cerebellar pathways in DS, suggesting cerebellar-driven mechanisms affecting mobility in DS (Lee et al., 2020).

However, these findings are in contrast with Romano et al. (2018), as well as with Fenoll et al. (2017), who reported microstructural integrity loss of the corticospinal tract in adults with DS as compared to healthy controls. Moreover, these authors observed integrity loss within the same sample and also in the SLF (see section “The Long-Association Fibres”), which is a key structure in the visuospatial integration and motor planning functions (Rushworth et al., 2006; De Schotten et al., 2011). These findings were interpreted by the authors as a neural sign of gait disturbance and motor deficits often reported in DS (Romano et al., 2018). To date, there is enough evidence to support both hypotheses made to account for the motor difficulties in DS (cerebellar-driven vs. corticospinal tract-driven mechanisms). However, it is important to note that Romano et al. (2018) used a more lenient threshold in their analysis, which could have potentially led to false positives. Future studies will shed light on the specific white matter-driven mechanism of motor impairments in young individuals with DS.

## Discussion

In this study, we reviewed all the available diffusion MRI investigations in populations with Down syndrome and discussed the implications of their findings in relation to Alzheimer’s disease. All these investigations represent a first attempt to characterise the *in-vivo* wiring of the brain in the population with DS. With this review, we aim to summarise and critically interpret the diffusion findings in DS to provide a useful starting point for future, hypothesis-driven, investigations using diffusion imaging in this DS.

What emerged from the examined studies is a picture of widespread integrity loss across a wide range of white matter tracts in DS (see Table 3). In particular, the frontal lobes appear to be the brain region with the most evident white matter differences as compared to controls in nearly all the investigations. This is in line with the structural MRI literature, in which volume loss of the frontal lobes is one of the most reported findings in DS and thought to be associated with the cognitive phenotype of poor executive function and working memory that is typical of this population (Grieco et al., 2015). Moreover, integrity loss of the frontal lobes is frequently observed in DsAD (for a review, see Fonseca et al., 2016). Indeed, personality and behavioural changes, which can potentially be traced back to the frontal domain, are amongst the first AD symptoms commonly reported in DS (Ball et al., 2006, 2008). These observations suggest a prominent frontal impact of AD neurodegeneration in DS. However, caution should be adopted in considering the frontal white matter integrity loss as a reliable indicator of the advent of AD in this population. More specifically, such frontally weighted neuron loss stands as a superimposition on a previously “less developed” brain area, in the context of which, even a relatively small additional loss due to AD will have a significant impact. Consequently, measures of neurodegeneration within the frontal lobes may possess low specificity in discriminating the AD from neurodevelopmental atypicalities.

**TABLE 3:** Main diffusion magnetic resonance imaging (MRI) findings in DS by age and diagnostic groups.

Age/Diagnostic groups	Mian diffusion MRI findings	References
Children/teenagers DS	Underdevelopment of projection fibres, such as anterior thalamic radiation, Internal capsule, cerebral and cerebellar peduncles, as well as pontine-cerebellar and olivo-cerebellar pathways. Furthermore, one study evidenced underdevelopment of the IFOF, ILF, UF.	Gunbey et al., 2017; Lee et al., 2020
Adult DS	All the main long association fibres and the corpus callosum are affected. Moreover, some studies evidenced reduced microstructural integrity of the anterior thalamic radiation and corticospinal tract.	Powell et al., 2014; Fenoll et al., 2017; Romano et al., 2018; Patrick et al., 2019; Bazydlo et al., 2021
DS with preclinical/prodromal AD	FA reduction of all the main long association fibres and the corpus callosum when compared to healthy adults with DS. FA of cingulum bundle, splenium of corpus callosum, ILF, and UF can distinguish DS with preclinical/prodromal AD from healthy DS	Rosas et al., 2020
DsAD	FA reduction of all the main long association fibres and the corpus callosum when compared to healthy adults with DS	Patrick et al., 2019; Rosas et al., 2020; Bazydlo et al., 2021

*DS, Down syndrome; DsAD, Down syndrome with Alzheimer disease; IFOF, inferior fronto-occipital fasciculus, ILF, inferior longitudinal fasciculus; SLF, superior longitudinal fasciculus; UF, uncinate fasciculus.*

The studies discussed in the present work examined individuals with DS belonging to several age groups, ranging from children with a mean age of 2.6 years (Gunbey et al., 2017) up to adults with DsAD aged 52 and above (Powell et al., 2014). While the prevalence of frontal lobes integrity loss appears to be common throughout the whole neurodevelopment in DS, some differences were observed at different development stages. Children and toddlers with DS tend to present with underdevelopment interesting mainly the projection fibres and cerebellar areas (Gunbey et al., 2017; Lee et al., 2020), while in adults and elderly individuals, the structural loss tends to affect the long association fibres and the corpus callosum (Powell et al., 2014; Fenoll et al., 2017; Romano et al., 2018; Rosas et al., 2020). However, these findings are preliminary given the few studies conducted so far.

A common finding observed virtually in all the investigations is the integrity loss of all the main long-association fibres in DS (see Figures 1, 2). The involvement of the long-association fibres is perhaps the main communality between sAD and DsAD. This group of bundles, also referred to as small-diameter fibres, myelinate much later during development (Yakovlev, 1967; Kinney et al., 1988) than the large-diameter fibre tracts, such as motor or sensory ones. According to the retrogenesis model, the progression of white matter degeneration in sAD reflects the reverse of the myelogenesis developmental order (Reisberg et al., 1999), with the late-myelination pathway being the first to be affected in sAD (Bartzokis, 2004). It is interesting to note that, despite the low number of DS diffusion examinations available, and despite most of these studies are on DS adults without AD, the white matter microstructure findings in DS seem to be in line with the retrogenesis model of sAD progression.

Finally, the cingulum bundle and the corpus callosum appear to be particularly relevant to the advent of AD in DS. FA of these two white matter structures (together with the ILF and UF) differed significantly between DS with preclinical/prodromal AD and healthy DS and were therefore considered sensitive to early AD-like changes (Rosas et al., 2020). The importance of the cingulum bundle is further highlighted by rs-fMRI studies, which point to a functional disconnection of the DMN posterior nodes in both sAD and DsAD (Badhwar et al., 2017; Wilson et al., 2019). On the other hand, integrity loss of the corpus callosum has been found in individuals with DS of all age groups. The corpus callosum is considered by some as a potential cognitive biomarker in this population, given its strong association with the DS cognitive phenotype (Teipel J. et al., 2003; Teipel and Hampel, 2006). Therefore, the cingulum bundle and the corpus callosum should be investigated in future diffusion studies of AD effects in DS.

## Limitations of Current Studies of White Matter Integrity in Down Syndrome

These preliminary studies represent a valuable starting point in understanding the white matter integrity in individuals with DS. However, data on the AD impact on white matter integrity are limited, because few studies included individuals with DsAD, and none employed a longitudinal design. Of these studies, Rosas et al. (2020) reported reduced FA of the SLF, UF, genu, splenium and cingulum bundle in DsAD, while Patrick et al. (2019) observed an increase in MD in IFOF and IFL. In contrast, no effect survived the correction for multiple comparisons in the study by Powell et al. (2014). Different issues may account for such heterogeneity in the results produced by these studies; first, it should be stressed that the population with DS manifests a wide degree of inter-individual heterogeneity of the syndrome’s manifestations, which unfolds at every level of description (e.g., genetic, cellular, cognitive, behavioural and environmental; Karmiloff-Smith et al., 2016). The age onset of the AD clinical manifestations is also variable, despite the AD histological feature being present in virtually 100% of this population (Strydom et al., 2018). Such heterogeneity, together with small sample sizes, might potentially dilute significant findings in cross-sectional studies that could be overcome by longitudinal study designs to improve our understanding of the AD development in DS.

An alternative explanation of different findings might be related to the peculiarities of diffusion metrics associated with aging. Fenoll et al. (2017) demonstrated an age-dependent effect for the reduction in FA in DS. However, this was also observed in the healthy control group, with no significant between-group difference in the strength of such an effect, thus failing to demonstrate the expected accelerated aging effect in DS. The authors speculated that FA was not sensitive enough to capture subtle age-related white matter alterations. Moreover, FA is a measure sensitive to noisy data (Seo et al., 2019), and evidence of DTI metrics other than FA having higher sensitivity in non-demented DS individuals has been reported (Romano et al., 2018; Patrick et al., 2019; Rosas et al., 2020). Axial diffusivity has been suggested to be a more suitable diffusion metric for discriminating MCI from healthy individuals in the general population (Agosta et al., 2011; Nir et al., 2013), and radial diffusivity can discriminate those brain regions in AD that were previously shown to be affected in MCI (Acosta-Cabronero et al., 2012).

Finally, Lee et al. (2020) suggested that FA may not be sensitive enough to the white matter differences in children and toddlers with DS compared to controls. The authors found that the volumetric properties of the white matter as obtained from alternative analytic diffusion approaches, such as tensor-based morphometry, could be more suited in examining the brain’s white matter in these age groups. This is because the diameter of smaller fibres could be determined by hypomyelination, which, in the absence of axonal structural damage, would only marginally affect anisotropy (Lee et al., 2020). Therefore, future studies should preferably include metrics other than FA, and consider alternative diffusion analytic approaches (e.g., Tensor-based morphometry; for a discussion see Dell’Acqua et al., 2013).

## Conclusion and Future Directions

In conclusion, what emerges from the present work is that diffusion imaging techniques constitute promising methods for the investigation of *in vivo* white matter development in DS and the impact of AD on neural connectivity. Moreover, metrics other than FA might prove sensitive in detecting early AD changes. Where possible, longitudinal designs should be implemented to address the inter-individual variability that characterise the population with DS. Longitudinal designs would also overcome the lack of standard reference for brain structures. As mentioned earlier, the brain of individuals with DS develops in an atypical fashion and with a high degree of interindividual variability. Several brain areas commonly affected in AD (i.e., temporal lobes) tend to be hypoplastic throughout the DS adulthood, making it difficult to discriminate AD-like neurodegenerative processes. This creates a situation in which there is a lack of a standard reference against which brain volume changes can be ascertained, rendering the AD diagnosis even more complicated.

A further layer of complexity is added by the fact that gross brain alterations observed *post-mortem* in sAD are considered non-specific in nature, as they show an extensive overlap with what is considered to be the product of healthy-ageing neural loss processes (Terry, 1986). Nevertheless, if severe brain volume alterations are observed before 65 years of age, using *in-vivo* volumetric methods, a diagnosis of AD can be made with a reasonable amount of confidence. In contrast, when considering the population with DS, the picture is more complicated due to the lack of a standard reference against which brain volume changes can be ascertained, given the background of atypical development, as well as the significant heterogeneity in clinical manifestation (Karmiloff-Smith et al., 2016).

The diffusion studies in DS discussed here adopted an explorative approach, employing voxel-wise analyses using small sample sizes. Despite that, lower levels of FA, as well as increased levels of MD affecting the main long association fibres and the corpus callosum have been reported by all these investigations when comparing individuals with DS with healthy controls (see Table 3). Therefore, future studies would benefit from employing a tractography approach, focusing on specific white matter structures, to obtain more detailed information that would normally elude voxel-wise analyses. In conclusion, diffusion imaging has the potential as a technique to identify biomarkers for the identification of the early stages of AD-related brain changes in the population with DS, particularly within the cingulum bundle and the corpus callosum. However, more studies are needed to validate the sensitivity and specificity of such measures as potential AD biomarkers.

## Author Contributions

FS, FD’A, and AS contributed to conception of the study. AS and FD’A supervised the project. FS carried out the literature search and wrote the manuscript with input from all authors. All authors contributed to the article and approved the submitted version.

## Conflict of Interest

The authors declare that the research was conducted in the absence of any commercial or financial relationships that could be construed as a potential conflict of interest.

## Publisher’s Note

All claims expressed in this article are solely those of the authors and do not necessarily represent those of their affiliated organizations, or those of the publisher, the editors and the reviewers. Any product that may be evaluated in this article, or claim that may be made by its manufacturer, is not guaranteed or endorsed by the publisher.

## References

[B1] Acosta-Cabronero J. Alley S. Williams G. B. Pengas G. Nestor P. J. (2012). Diffusion tensor metrics as biomarkers in Alzheimer’s disease. *PLoS One* 7:e49072. 10.1371/journal.pone.0049072 23145075 PMC3492261

[B2] Agosta F. Pievani M. Sala S. Geroldi C. Galluzzi S. Frisoni G. B. (2011). White matter damage in Alzheimer disease and its relationship to gray matter atrophy. *Radiology* 258 853–863. 21177393 10.1148/radiol.10101284

[B3] Almairac F. Herbet G. Moritz-Gasser S. De Champfleur N. M. Duffau H. (2015). The left inferior fronto-occipital fasciculus subserves language semantics: a multilevel lesion study. *Brain Struct. Funct.* 220 1983–1995. 24744151 10.1007/s00429-014-0773-1

[B4] Alves G. S. O’dwyer L. Jurcoane A. Oertel-Knöchel V. Knöchel C. Prvulovic D. (2012). Different patterns of white matter degeneration using multiple diffusion indices and volumetric data in mild cognitive impairment and Alzheimer patients. *PLoS One* 7:e52859. 10.1371/journal.pone.0052859 23300797 PMC3534120

[B5] Anderson J. S. Nielsen J. A. Ferguson M. A. Burback M. C. Cox E. T. Dai L. (2013). Abnormal brain synchrony in Down syndrome. *NeuroImage* 2 703–715. 24179822 10.1016/j.nicl.2013.05.006 PMC3778249

[B6] Andrews-Hanna J. R. Smallwood J. Spreng R. N. (2014). The default network and self-generated thought: component processes, dynamic control, and clinical relevance. *Ann. N. Y. Acad. Sci.* 1316 29–52. 10.1111/nyas.12360 24502540 PMC4039623

[B7] Annus T. Wilson L. R. Acosta-Cabronero J. Cardenas-Blanco A. Hong Y. T. Fryer T. D. (2017). The Down syndrome brain in the presence and absence of fibrillar β-amyloidosis. *Neurobiol. Aging* 53 11–19. 10.1016/j.neurobiolaging.2017.01.009 28192686 PMC5391869

[B8] Antonarakis S. E. Skotko B. G. Rafii M. S. Strydom A. Pape S. E. Bianchi D. W. (2020). Down syndrome. *Nat. Rev. Dis. Primers* 6:9. 10.1038/s41572-019-0143-7 PMC8428796 32029743

[B9] Badhwar A. Tam A. Dansereau C. Orban P. Hoffstaedter F. Bellec P. (2017). Resting-state network dysfunction in Alzheimer’s disease: a systematic review and meta-analysis. *Alzheimer’s Dement.* 8 73–85. 10.1016/j.dadm.2017.03.007 PMC5436069 28560308

[B10] Ball S. L. Holland A. J. Hon J. Huppert F. A. Treppner P. Watson P. C. (2006). Personality and behaviour changes mark the early stages of Alzheimer’s disease in adults with Down’s syndrome: findings from a prospective population-based study. *Int. J. Geriatr. Psychiatry* 21 661–673. 10.1002/gps.1545 16802281

[B11] Ball S. L. Holland A. J. Treppner P. Watson P. C. Huppert F. A. (2008). Executive dysfunction and its association with personality and behaviour changes in the development of Alzheimer’s disease in adults with Down syndrome and mild to moderate learning disabilities. *Br. J. Clin. Psychol.* 47 1–29. 10.1348/014466507X230967 17681112

[B12] Bartzokis G. (2004). Age-related myelin breakdown: a developmental model of cognitive decline and Alzheimer’s disease. *Neurobiol. Aging* 25 5–18. 14675724 10.1016/j.neurobiolaging.2003.03.001

[B13] Basser P. J. Mattiello J. Lebihan D. (1994). MR diffusion tensor spectroscopy and imaging. *Biophys. J.* 66 259–267. 8130344 10.1016/S0006-3495(94)80775-1 PMC1275686

[B14] Bazydlo A. Zammit M. Wu M. Dean D. Johnson S. Tudorascu D. (2021). White matter microstructure associations with episodic memory in adults with Down syndrome: a tract-based spatial statistics study. *J. Neurodev. Disord.* 13:17. 10.1186/s11689-021-09366-1 33879062 PMC8059162

[B15] Beacher F. Daly E. Simmons A. Prasher V. Morris R. Robinson C. (2009). Alzheimer’s disease and Down’s syndrome: an *in vivo* MRI study. *Psychol. Med.* 39 675–684. 10.1017/S0033291708004054 18667098

[B16] Bletsch A. Mann C. Andrews D. S. Daly E. Tan G. M. Murphy D. G. (2018). Down syndrome is accompanied by significantly reduced cortical grey–white matter tissue contrast. *Hum. Brain Mapp.* 39 4043–4054. 10.1002/hbm.24230 29885016 PMC6866483

[B17] Bosch B. Arenaza-Urquijo E. M. Rami L. Sala-Llonch R. Junqué C. Solé-Padullés C. (2012). Multiple Dti index analysis in normal aging, amnestic MCI and AD. Relationship with neuropsychological performance. *Neurobiol. Aging* 33 61–74. 10.1016/j.neurobiolaging.2010.02.004 20371138

[B18] Bubb E. J. Metzler-Baddeley C. Aggleton J. P. (2018). The cingulum bundle: anatomy, function, and dysfunction. *Neurosci. Biobehav. Rev.* 92 104–127. 29753752 10.1016/j.neubiorev.2018.05.008 PMC6090091

[B19] Buckner R. L. Sepulcre J. Talukdar T. Krienen F. M. Liu H. Hedden T. (2009). Cortical hubs revealed by intrinsic functional connectivity: mapping, assessment of stability, and relation to Alzheimer’s disease. *J. Neurosci.* 29 1860–1873. 10.1523/JNEUROSCI.5062-08.2009 19211893 PMC2750039

[B20] Carducci F. Onorati P. Condoluci C. Di Gennaro G. Quarato P. P. Pierallini A. (2013). Whole-brain voxel-based morphometry study of children and adolescents with Down syndrome. *Funct. Neurol.* 28:19. PMC3812718 23731912

[B21] Catani M. Dawson M. (2017). “Language processing, development and evolution,” in *Conn’s Translational Neuroscience*, ed. Michael Conn P. (Amsterdam: Elsevier), 679–692.

[B22] Catani M. De Schotten M. T. (2008). A diffusion tensor imaging tractography atlas for virtual *in vivo* dissections. *Cortex* 44 1105–1132. 18619589 10.1016/j.cortex.2008.05.004

[B23] Catani M. De Schotten M. T. (2012). *Atlas Of Human Brain Connections.* Oxford: Oxford University Press.

[B24] Catani M. Howard R. J. Pajevic S. Jones D. K. (2002). Virtual *in vivo* interactive dissection of white matter fasciculi in the human brain. *Neuroimage* 17 77–94. 10.1006/nimg.2002.1136 12482069

[B25] Davidson Y. S. Robinson A. Prasher V. P. Mann D. (2018). The age of onset and evolution of Braak tangle stage and Thal amyloid pathology of Alzheimer’s disease in individuals with Down syndrome. *Acta Neuropathol. Commun.* 6:56. 10.1186/s40478-018-0559-4 29973279 PMC6030772

[B26] De Schotten M. T. Dell’acqua F. Forkel S. Simmons A. Vergani F. Murphy D. G. (2011). A lateralized brain network for visuo-spatial attention. *Nat. Preced.* 14 1245–1246. 10.1038/nn.2905 21926985

[B27] Dekker A. D. Strydom A. Coppus A. M. Nizetic D. Vermeiren Y. Naudé P. J. (2015). Behavioural and psychological symptoms of dementia in Down syndrome: early indicators of clinical Alzheimer’s disease? *Cortex* 73 36–61. 26343344 10.1016/j.cortex.2015.07.032

[B28] Dell’Acqua F. Simmons A. Williams S. C. Catani M. (2013). Can spherical deconvolution provide more information than fiber orientations? Hindrance modulated orientational anisotropy, a true-tract specific index to characterize white matter diffusion. *Hum. Brain Mapp.* 34 2464–2483. 10.1002/hbm.22080 22488973 PMC6870506

[B29] Duffau H. (2008). The anatomo-functional connectivity of language revisited: new insights provided by electrostimulation and tractography. *Neuropsychologia* 46 927–934. 10.1016/j.neuropsychologia.2007.10.025 18093622

[B30] Duffau H. Herbet G. Moritz-Gasser S. (2013). Toward a pluri-component, multimodal, and dynamic organization of the ventral semantic stream in humans: lessons from stimulation mapping in awake patients. *Front. Syst. Neurosci.* 7:44. 10.3389/fnsys.2013.00044 23986661 PMC3752437

[B31] Fenoll R. Pujol J. Esteba-Castillo S. De Sola S. Ribas-Vidal N. García-Alba J. (2017). Anomalous white matter structure and the effect of age in Down syndrome patients. *J. Alzheimer’s Dis.* 57 61–70. 10.3233/JAD-161112 28222523

[B32] Figueroa-Jimenez M. D. Carbó-Carreté M. Cañete-Massé C. Zarabozo-Hurtado D. Peró-Cebollero M. Salazar-Estrada J. G. (2021). Complexity Analysis of the Default Mode Network Using Resting-State fMRI in Down Syndrome: relationships Highlighted by a Neuropsychological Assessment. *Brain Sci.* 11:311. 10.3390/brainsci11030311 33801471 PMC8001398

[B33] Fonseca L. M. Yokomizo J. E. Bottino C. M. Fuentes D. (2016). Frontal lobe degeneration in adults with Down syndrome and Alzheimer’s disease: a review. *Dement. Geriatr. Cogn. Dis.* 41 123–136. 10.1159/000442941 26891227

[B34] Fox M. D. Corbetta M. Snyder A. Z. Vincent J. L. Raichle M. E. (2006). Spontaneous neuronal activity distinguishes human dorsal and ventral attention systems. *Proc. Natl. Acad. Sci.U.S.A.* 103 10046–10051. 10.1073/pnas.0604187103 16788060 PMC1480402

[B35] Fox M. D. Snyder A. Z. Vincent J. L. Corbetta M. Van Essen D. C. Raichle M. E. (2005). The human brain is intrinsically organized into dynamic, anticorrelated functional networks. *Proc. Natl. Acad. Sci.U.S.A.* 102 9673–9678. 15976020 10.1073/pnas.0504136102 PMC1157105

[B36] Fransson P. (2006). How default is the default mode of brain function?: further evidence from intrinsic BOLD signal fluctuations. *Neuropsychologia* 44 2836–2845. 16879844 10.1016/j.neuropsychologia.2006.06.017

[B37] Fransson P. Marrelec G. (2008). The precuneus/posterior cingulate cortex plays a pivotal role in the default mode network: evidence from a partial correlation network analysis. *Neuroimage* 42 1178–1184. 10.1016/j.neuroimage.2008.05.059 18598773

[B38] Greicius M. D. Krasnow B. Reiss A. L. Menon V. (2003). Functional connectivity in the resting brain: a network analysis of the default mode hypothesis. *Proc. Natl. Acad. Sci.U.S.A.* 100 253–258. 10.1073/pnas.0135058100 12506194 PMC140943

[B39] Grieco J. Pulsifer M. Seligsohn K. Skotko B. Schwartz A. (2015). Down syndrome: cognitive and behavioral functioning across the lifespan. *Am. J. Med. Genet. Part C* 169 135–149. 25989505 10.1002/ajmg.c.31439

[B40] Gunbey H. P. Bilgici M. C. Aslan K. Has A. C. Ogur M. G. Alhan A. (2017). Structural brain alterations of Down’s syndrome in early childhood evaluation by DTI and volumetric analyses. *Eur. Radiol.* 27 3013–3021. 10.1007/s00330-016-4626-6 27798752

[B41] Gusnard D. A. Raichle M. E. (2001). Searching for a baseline: functional imaging and the resting human brain. *Nat. Rev. Neurosci.* 2 685–694. 11584306 10.1038/35094500

[B42] Heath M. Welsh T. N. Simon D. A. Tremblay L. Elliott D. Roy E. A. (2005). Relative processing demands influence cerebral laterality for verbal-motor integration in persons with Down syndrome. *Cortex* 41 61–66. 10.1016/s0010-9452(08)70178-3 15633707

[B43] Herbet G. Zemmoura I. Duffau H. (2018). Functional anatomy of the inferior longitudinal fasciculus: from historical reports to current hypotheses. *Front. Neuroanat.* 12:77. 10.3389/fnana.2018.00077 30283306 PMC6156142

[B44] Hillary F. G. Grafman J. H. (2017). Injured brains and adaptive networks: the benefits and costs of hyperconnectivity. *Trends Cogn. Sci.* 21 385–401. 10.1016/j.tics.2017.03.003 28372878 PMC6664441

[B45] Huang H. Fan X. Weiner M. Martin-Cook K. Xiao G. Davis J. (2012). Distinctive disruption patterns of white matter tracts in Alzheimer’s disease with full diffusion tensor characterization. *Neurobiol. Aging* 33 2029–2045. 10.1016/j.neurobiolaging.2011.06.027 21872362 PMC3227739

[B46] Ismail Z. Smith E. E. Geda Y. Sultzer D. Brodaty H. Smith G. (2016). Neuropsychiatric symptoms as early manifestations of emergent dementia: provisional diagnostic criteria for mild behavioral impairment. *Alzheimer’s Demen.* 12 195–202. 10.1016/j.jalz.2015.05.017 26096665 PMC4684483

[B47] Jacobs H. I. Radua J. Lückmann H. C. Sack A. T. (2013). Meta-analysis of functional network alterations in Alzheimer’s disease: toward a network biomarker. *Neurosci. Biobehav. Rev.* 37 753–765. 10.1016/j.neubiorev.2013.03.009 23523750

[B48] Jones D. K. Knösche T. R. Turner R. (2013). White matter integrity, fiber count, and other fallacies: the do’s and don’ts of diffusion MRI. *Neuroimage* 73 239–254. 10.1016/j.neuroimage.2012.06.081 22846632

[B49] Karmiloff-Smith A. Al-Janabi T. D’souza H. Groet J. Massand E. Mok K. (2016). The importance of understanding individual differences in Down syndrome. *F1000Res.* 5:F1000FacultyRev–389. 10.12688/f1000research.7506.1 PMC4806704 27019699

[B50] Kelly A. C. Uddin L. Q. Biswal B. B. Castellanos F. X. Milham M. P. (2008). Competition between functional brain networks mediates behavioral variability. *Neuroimage* 39 527–537. 17919929 10.1016/j.neuroimage.2007.08.008

[B51] Kinney H. C. Brody B. A. Kloman A. S. Gilles F. H. (1988). Sequence of central nervous system myelination in human infancy: ii. Patterns of myelination in autopsied infants. *J. Neuropathol. Exp. Neurol.* 47 217–234. 10.1097/00005072-198805000-00003 3367155

[B52] Koenig K. A. Ruedrich S. Bekris L. M. Khrestian M. Kim S. Leverenz J. B. (2019). White matter integrity and inflammation at 7 tesla in adults with Down syndrome. *Alzheimer’s Dement.* 15:951.

[B53] Kwon H. Hong J. Jang S. (2011). Anatomic location and somatotopic arrangement of the corticospinal tract at the cerebral peduncle in the human brain. *Am. J. Neuroradiol.* 32 2116–2119. 10.3174/ajnr.A2660 21903908 PMC7964417

[B54] Lao P. J. Handen B. L. Betthauser T. J. Mihaila I. Hartley S. L. Cohen A. D. (2017). Longitudinal changes in amyloid positron emission tomography and volumetric magnetic resonance imaging in the nondemented Down syndrome population. *Alzheimer’s Dement.* 9 1–9. 10.1016/j.dadm.2017.05.001 PMC5454131 28603769

[B55] Lee N. R. Adeyemi E. I. Lin A. Clasen L. S. Lalonde F. M. Condon E. (2015). Dissociations in cortical morphometry in youth with Down syndrome: evidence for reduced surface area but increased thickness. *Cereb. Cortex* 26 2982–2990. 10.1093/cercor/bhv107 26088974 PMC4898663

[B56] Lee N. R. Nayak A. Irfanoglu M. O. Sadeghi N. Stoodley C. J. Adeyemi E. (2020). Hypoplasia of cerebellar afferent networks in Down syndrome revealed by DTI-driven tensor based morphometry. *Sci. Rep.* 10:5447. 10.1038/s41598-020-61799-1 32214129 PMC7096514

[B57] Mamah D. Conturo T. E. Harms M. P. Akbudak E. Wang L. Mcmichael A. R. (2010). Anterior thalamic radiation integrity in schizophrenia: a diffusion-tensor imaging study. *Psychiatry Res.* 183 144–150. 20619618 10.1016/j.pscychresns.2010.04.013 PMC3887223

[B58] McCarron M. Mccallion P. Reilly E. Dunne P. Carroll R. Mulryan N. (2017). A prospective 20-year longitudinal follow-up of dementia in persons with Down syndrome. *J. Intellect. Disabil. Res.* 61 843–852. 10.1111/jir.12390 28664561

[B59] Menghini D. Costanzo F. Vicari S. (2011). Relationship between brain and cognitive processes in Down syndrome. *Behav. Genet.* 41 381–393. 21279430 10.1007/s10519-011-9448-3

[B60] Nestor P. G. Kubicki M. Gurrera R. J. Niznikiewicz M. Frumin M. Mccarley R. W. (2004). Neuropsychological correlates of diffusion tensor imaging in schizophrenia. *Neuropsychology* 18:629. 10.1037/0894-4105.18.4.629 PMC2790923 15506830

[B61] Nir T. M. Jahanshad N. Villalon-Reina J. E. Toga A. W. Jack C. R. Weiner M. W. (2013). Effectiveness of regional Dti measures in distinguishing Alzheimer’s disease. MCI, and normal aging. *NeuroImage* 3 180–195. 10.1016/j.nicl.2013.07.006 24179862 PMC3792746

[B62] Parker S. E. Mai C. T. Canfield M. A. Rickard R. Wang Y. Meyer R. E. (2010). Updated national birth prevalence estimates for selected birth defects in the United States, 2004–2006. *Birth Defects Res. Part A* 88 1008–1016. 10.1002/bdra.20735 20878909

[B63] Patrick A. Wu M. Lao P. J. Dean Iii D. C. Zammit M. D. Johnson S. C. (2019). P3-325: amyloid-B Associations With White Matter In Down Syndrome Assessed Using Tract-Based Spatial Statistics (Tbss) And 11c-Pib Positron Emission Tomography. *Alzheimer’s Dement.* 15 1063–1063.

[B64] Pierpaoli C. Basser P. J. (1996). Toward a quantitative assessment of diffusion anisotropy. *Magn. Reson. Med.* 36 893–906. 10.1002/mrm.1910360612 8946355

[B65] Pini L. Pievani M. Bocchetta M. Altomare D. Bosco P. Cavedo E. (2016). Brain atrophy in Alzheimer’s disease and aging. *Ageing Res. Rev.* 30 25–48. 26827786 10.1016/j.arr.2016.01.002

[B66] Pinter J. D. Eliez S. Schmitt J. E. Capone G. T. Reiss A. L. (2001). Neuroanatomy of Down’s syndrome: a high-resolution MRI study. *Am. J. Psychiatry* 158 1659–1665. 11578999 10.1176/appi.ajp.158.10.1659

[B67] Powell D. Caban-Holt A. Jicha G. Robertson W. Davis R. Gold B. T. (2014). Frontal white matter integrity in adults with Down syndrome with and without dementia. *Neurobiol. Aging* 35 1562–1569. 10.1016/j.neurobiolaging.2014.01.137 24582640 PMC3992921

[B68] Pujol J. Fenoll R. Ribas-Vidal N. Martínez-Vilavella G. Blanco-Hinojo L. García-Alba J. (2018). A longitudinal study of brain anatomy changes preceding dementia in Down syndrome. *NeuroImage* 18 160–166. 10.1016/j.nicl.2018.01.024 29868444 PMC5984600

[B69] Raybaud C. (2010). The corpus callosum, the other great forebrain commissures, and the septum pellucidum: anatomy, development, and malformation. *Neuroradiology* 52 447–477. 10.1007/s00234-010-0696-3 20422408

[B70] Reisberg B. Franssen E. H. Hasan S. M. Monteiro I. Boksay I. Souren L. E. (1999). Retrogenesis: clinical, physiologic, and pathologic mechanisms in brain aging, Alzheimer’s and other dementing processes. *Eur. Arch. Psychiatry Clin. Neurosci.* 249 S28–S36. 10.1007/pl00014170 10654097

[B71] Romano A. Moraschi M. Cornia R. Bozzao A. Rossi-Espagnet M. C. Giove F. (2018). White matter involvement in young non-demented Down’s syndrome subjects: a tract-based spatial statistic analysis. *Neuroradiology* 60 1335–1341. 10.1007/s00234-018-2102-5 30264168

[B72] Rosas H. D. Hsu E. Mercaldo N. D. Lai F. Pulsifer M. Keator D. (2020). Alzheimer-related altered white matter microstructural integrity in Down syndrome: a model for sporadic AD? *Alzheimer’s Dement.* 12:e12040. 10.1002/dad2.12040 33204811 PMC7648416

[B73] Rushworth M. Behrens T. Johansen-Berg H. (2006). Connection patterns distinguish 3 regions of human parietal cortex. *Cereb. Cortex* 16 1418–1430. 16306320 10.1093/cercor/bhj079

[B74] Sabbagh M. N. Chen K. Rogers J. Fleisher A. S. Liebsack C. Bandy D. (2015). Florbetapir PET, FDG PET, and MRI in Down syndrome individuals with and without Alzheimer’s dementia. *Alzheimer’s Dement.* 11 994–1004. 10.1016/j.jalz.2015.01.006 25849033 PMC4543530

[B75] Schmahmann J. D. Pandya D. N. Wang R. Dai G. D’arceuil H. E. De Crespigny A. J. (2007). Association fibre pathways of the brain: parallel observations from diffusion spectrum imaging and autoradiography. *Brain* 130 630–653. 10.1093/brain/awl359 17293361

[B76] Seo Y. Rollins N. K. Wang Z. J. (2019). Reduction of bias in the evaluation of fractional anisotropy and mean diffusivity in magnetic resonance diffusion tensor imaging using region-of-interest methodology. *Sci. Rep.* 9:13095. 10.1038/s41598-019-49311-w 31511553 PMC6739503

[B77] Shoji H. Koizumi N. Ozaki H. (2009). Linguistic lateralization in adolescents with Down syndrome revealed by a dichotic monitoring test. *Res. Dev. Disabil.* 30 219–228. 10.1016/j.ridd.2008.03.004 18482829

[B78] Solowij N. Zalesky A. Lorenzetti V. Yücel M. (2017). “Chapter 40 - Chronic Cannabis Use and Axonal Fiber Connectivity,” in *Handbook of Cannabis and Related Pathologies*, ed. PREEDY V. R. (San Diego: Academic Press).

[B79] Squire L. R. Zola-Morgan S. (1991). The medial temporal lobe memory system. *Science* 253 1380–1386. 1896849 10.1126/science.1896849

[B80] Stricker N. H. Schweinsburg B. Delano-Wood L. Wierenga C. E. Bangen K. J. Haaland K. (2009). Decreased white matter integrity in late-myelinating fiber pathways in Alzheimer’s disease supports retrogenesis. *Neuroimage* 45 10–16. 10.1016/j.neuroimage.2008.11.027 19100839 PMC2782417

[B81] Strydom A. Coppus A. Blesa R. Danek A. Fortea J. Hardy J. (2018). Alzheimer’s disease in Down syndrome: an overlooked population for prevention trials. *Alzheimer’s Dement.* 4 703–713. 10.1016/j.trci.2018.10.006 30581976 PMC6296162

[B82] Takahashi S. Yonezawa H. Takahashi J. Kudo M. Inoue T. Tohgi H. (2002). Selective reduction of diffusion anisotropy in white matter of Alzheimer disease brains measured by 3.0 Tesla magnetic resonance imaging. *Neurosci. Lett.* 332 45–48. 10.1016/s0304-3940(02)00914-x 12377381

[B83] Taoka T. Morikawa M. Akashi T. Miyasaka T. Nakagawa H. Kiuchi K. (2009). Fractional anisotropy–threshold dependence in tract-based diffusion tensor analysis: evaluation of the uncinate fasciculus in Alzheimer disease. *Am. J. Neuroradiol.* 30 1700–1703. 10.3174/ajnr.A1698 19541775 PMC7051508

[B84] Teipel J. Bayer W. Alexander G. Bokde A. Zebuhr Y. Teichberg D. (2003). Regional pattern of hippocampus and corpus callosum atrophy in Alzheimer’s disease in relation to dementia severity: evidence for early neocortical degeneration. *Neurobiol. Aging* 24 85–94. 12493554 10.1016/s0197-4580(02)00044-1

[B85] Teipel S. J. Alexander G. E. Schapiro M. B. Möller H. J. Rapoport S. I. Hampel H. (2004). Age-related cortical grey matter reductions in non-demented Down’s syndrome adults determined by MRI with voxel-based morphometry. *Brain* 127 811–824. 10.1093/brain/awh101 14985261

[B86] Teipel S. J. Hampel H. (2006). Neuroanatomy of Down syndrome *in vivo*: a model of preclinical Alzheimer’s disease. *Behav. Genet.* 36 405–415. 10.1007/s10519-006-9047-x 16485178

[B87] Teipel S. J. Schapiro M. B. Alexander G. E. Krasuski J. S. Horwitz B. Hoehne C. (2003). Relation of corpus callosum and hippocampal size to age in nondemented adults with Down’s syndrome. *Am. J. Psychiatry* 160 1870–1878. 10.1176/appi.ajp.160.10.1870 14514503

[B88] Terry R. D. (1986). Interrelations among the lesions of normal and abnormal aging of the brain. *Prog. Brain Res.* 70 41–48. 10.1016/s0079-6123(08)64296-x 3554358

[B89] van der Knaap L. J. van der Ham I. J. (2011). How does the corpus callosum mediate interhemispheric transfer? A review. *Behav. Brain Res.* 223 211–221. 21530590 10.1016/j.bbr.2011.04.018

[B90] Vega J. N. Hohman T. J. Pryweller J. R. Dykens E. M. Thornton-Wells T. A. (2015). Resting-state functional connectivity in individuals with Down syndrome and Williams syndrome compared with typically developing controls. *Brain Connect.* 5 461–475. 25712025 10.1089/brain.2014.0266 PMC4601631

[B91] Wang K. Liang M. Wang L. Tian L. Zhang X. Li K. (2007). Altered functional connectivity in early Alzheimer’s disease: a resting-state fMRI study. *Hum. Brain Mapp.* 28 967–978. 17133390 10.1002/hbm.20324 PMC6871392

[B92] Weiler M. De Campos B. M. De Ligo Teixeira C. V. Casseb R. F. Carletti-Cassani A. F. M. K. Vicentini J. E. (2017). Intranetwork and internetwork connectivity in patients with Alzheimer disease and the association with cerebrospinal fluid biomarker levels. *J. Psychiatry Neurosci.* 42 366–377. 28375076 10.1503/jpn.160190 PMC5662458

[B93] Weissman D. H. Roberts K. Visscher K. Woldorff M. (2006). The neural bases of momentary lapses in attention. *Nat. Neurosci.* 9 971–978. 16767087 10.1038/nn1727

[B94] White N. S. Alkire M. T. Haier R. J. (2003). A voxel-based morphometric study of nondemented adults with Down Syndrome. *Neuroimage* 20 393–403. 10.1016/s1053-8119(03)00273-8 14527599

[B95] Wilson L. R. Vatansever D. Annus T. Williams G. B. Hong Y. T. Fryer T. D. (2019). Differential effects of Down’s syndrome and Alzheimer’s neuropathology on default mode connectivity. *Hum. Brain Mapp.* 40 4551–4563. 10.1002/hbm.24720 31350817 PMC6865660

[B96] Witelson S. F. (1989). Hand and sex differences in the isthmus and genu of the human corpus callosum: a postmortem morphological study. *Brain* 112 799–835. 10.1093/brain/112.3.799 2731030

[B97] Wu Y. Sun D. Wang Y. Wang Y. (2016). Subcomponents and connectivity of the inferior fronto-occipital fasciculus revealed by diffusion spectrum imaging fiber tracking. *Front. Neuroanat.* 10:88. 10.3389/fnana.2016.00088 27721745 PMC5033953

[B98] Yakovlev P. (1967). “The myelogenetic cycles of regional maturation of the brain,” in *Regional Development Of The Brain In Early Life*, ed. Minkowski A. (Hoboken: Blackwell Science), 3–70. 10.1007/BF00192215

[B99] Zhang Y. Schuff N. Jahng G.-H. Bayne W. Mori S. Schad L. (2007). Diffusion tensor imaging of cingulum fibers in mild cognitive impairment and Alzheimer disease. *Neurology* 68 13–19. 17200485 10.1212/01.wnl.0000250326.77323.01 PMC1941719

[B100] Zhang Y. Yang J. Jiang W. Li R. Zhu M. Xiong F. (2019). Brain Development Measured With MRI in Children With Down Syndrome Correlates With Blood Biochemical Biomarkers. *Pediatr. Neurol.* 92 43–47. 10.1016/j.pediatrneurol.2018.10.007 30612744

